# The epigenetic clock and physical development during childhood and adolescence: longitudinal analysis from a UK birth cohort

**DOI:** 10.1093/ije/dyw307

**Published:** 2017-01-15

**Authors:** Andrew J Simpkin, Laura D Howe, Kate Tilling, Tom R Gaunt, Oliver Lyttleton, Wendy L McArdle, Susan M Ring, Steve Horvath, George Davey Smith, Caroline L Relton

**Affiliations:** 1 MRC Integrative Epidemiology Unit, School of Social and Community Medicine, University of Bristol, Bristol, UK; 2 School of Social and Community Medicine, University of Bristol, Bristol, UK; 3 School of Public Health, University of California Los Angeles, Los Angeles, CA, USA; 4 David Geffen School of Medicine, University of California Los Angeles, Los Angeles, CA, USA

**Keywords:** ALSPAC, ARIES, DNA methylation, epigenetic age, longitudinal data analysis, physical development

## Abstract

**Background:** Statistical models that use an individual’s DNA methylation levels to estimate their age (known as epigenetic clocks) have recently been developed, with 96% correlation found between epigenetic and chronological age. We postulate that differences between estimated and actual age [age acceleration (AA)] can be used as a measure of developmental age in early life.

**Methods:** We obtained DNA methylation measures at three time points (birth, age 7 years and age 17 years) in 1018 children from the Avon Longitudinal Study of Parents and Children (ALSPAC). Using an online calculator, we estimated epigenetic age, and thus AA, for each child at each time point. We then investigated whether AA was prospectively associated with repeated measures of height, weight, body mass index (BMI), bone mineral density, bone mass, fat mass, lean mass and Tanner stage.

**Results:** Positive AA at birth was associated with higher average fat mass [1321 g per year of AA, 95% confidence interval (CI) 386, 2256 g] from birth to adolescence (i.e. from age 0–17 years) and AA at age 7 was associated with higher average height (0.23 cm per year of AA, 95% CI 0.04, 0.41 cm). Conflicting evidence for the role of AA (at birth and in childhood) on changes during development was also found, with higher AA being positively associated with changes in weight, BMI and Tanner stage, but negatively with changes in height and fat mass.

**Conclusions:** We found evidence that being ahead of one’s epigenetic age acceleration is related to developmental characteristics during childhood and adolescence. This demonstrates the potential for using AA as a measure of development in future research.

Key Messages

- Children with a positive epigenetic age are taller and have higher fat mass throughout childhood and adolescence on average.
- Epigenetic age acceleration is associated with longitudinal changes in weight, BMI, height and fat mass during childhood and adolescence.
- We find some evidence that higher epigenetic age is positively associated with longitudinal Tanner stage of development in adolescents.
- We find no association between epigenetic age and age at puberty, estimated as the age at peak height velocity.

## Introduction

Statistical models that use an individual’s DNA methylation levels to estimate their age (known as epigenetic clocks) have been developed.^1–5^ These methods have proved successful, with up to 96% correlation and a mean difference of 3 years found between estimated and actual age.^2^ A recent review^6^ has also highlighted two separate processes when it comes to age-related changes of DNA methylation levels: one reflecting overall changes in DNA methylation across CpG sites over the life course (sometimes referred to as epigenetic drift^7–9^), which may be attributed to individual level environmental factors or stochastic processes. The second uses specific CpG sites that are affected by age in a similar fashion across individuals, and hence can be used to accurately predict age from DNA methylation data. Differences between chronological age and epigenetic age are defined as age acceleration (AA) and positive age acceleration (i.e. having a higher epigenetic age than chronological age) has been shown to be associated with obesity,^10^ lower physical and cognitive function,^11^ Alzheimer’s disease,^12^ HIV,^13^ menopause^14^ and all-cause mortality.^15–17^ Since DNA methylation can be influenced by environmental factors,^18^ and in turn influence phenotypes, it is of interest to study both the determinants and consequences of AA. However, there is an absence of literature on the associations of AA with physical development in early life. The Avon Longitudinal Study of Parents and Children (ALSPAC)^19^^,^^20^ is a large UK birth cohort, which has followed roughly 14 000 children from birth, collecting many thousands of variables over time. DNA methylation data were obtained for 1018 of these children from umbilical cord blood (at birth) and venous blood at ages 7 and 15 or 17 years as part of the Accessible Resource for Integrated Epigenomic Studies (ARIES) project.^21^

Here we use the epigenetic clock method by Horvath, for the following reasons: first, it is more accurate than other methods when it comes to young subjects^22^^,^^23^; second, it applies to virtually all tissues and cell types, which suggests that it might play a role in organismal development and ageing. Using the Horvath age estimation method, we have calculated the epigenetic age for all of the children at each time point, and the resulting AA. In this paper, we investigate the consequences of AA, by looking at standard measures of development, which have been repeatedly measured throughout childhood and adolescence: height, weight, body mass index (BMI), bone mineral density (BMD), bone mass, lean mass and fat mass.

## Methods

### Study population

This study used DNA methylation data generated under the auspices of the ALSPAC.^19^^,^^20^ ALSPAC recruited 14 541 pregnant women with expected delivery dates between April 1991 and December 1992. Of these initial pregnancies, there were 14 062 live births and 13 988 children who were alive at 1 year of age. The study website contains details of all the data that are available through a fully searchable data dictionary (http://www.bris.ac.uk/alspac/researchers/data-access/data-dictionary).

As part of the ARIES^21^ project (http://www.ariesepigenomics.org.uk), a sub-sample of 1018 ALSPAC mother–child pairs had DNA methylation measured using the Infinium HumanMethylation450 BeadChip (Illumina, Inc.)^24^ Here, we use DNA methylation data generated from cord blood and venous blood samples at age 7 and again at age 15 or 17 years, leading to three measurements of DNA methylation per child. All DNA methylation wet-lab and preprocessing analyses were performed at the University of Bristol as part of the ARIES project and has been described in detail previously.^21^^,^^22^

### Epigenetic age

Using the online epigenetic clock calculator (http://labs.genetics.ucla.edu/horvath/dnamage/), we obtained epigenetic age for each child at each time point in ARIES. Along with epigenetic age, the online calculator estimates cell-type proportions and calculates raw AA differences (estimated chronological age) and AA residuals (the residuals from a linear regression of epigenetic age on chronological age, which we call age acceleration and denote as ‘AA’). These AAs are uncorrelated with chronological age and contain information about the epigenetic age profiles of each sample, i.e. a positive residual corresponds to an individual whose epigenetic age is ahead of their chronological age and vice versa. The calculator provides estimates of epigenetic age, AA and AA adjusted for imputed blood cell types. In our analysis, we use those AA residuals which have been adjusted for estimated cell-type ratios.

### Developmental variables

We obtained longitudinal data on repeatedly measured physical characteristics in ALSPAC to investigate the relationship between AA and development. These characteristics were height (cm), weight (kg), BMI (kg/m^2^), BMD (g/cm^2^), bone mass (g), fat mass (g) and lean mass (g). Height, weight and BMI were measured from birth to age 18 years, with up to 19 measurements per child, including nine after age 7 years; BMD, bone mass, fat mass and lean mass were assessed by dual energy X-ray absorptiometry (DXA) scans twice, at ages 9 and 18 years. Age at puberty was estimated by age at peak height velocity (PHV)^25^ calculated using the SITAR model.^26^ We included estimated age at puberty in all longitudinal models of development and also investigated whether it was related to AA. Tanner (25) staging was repeatedly measured at mean ages 8.2, 9.7, 10.8, 11.8, 13.2 and 14.7 years. At each of these six ages, participants were asked to mark their development in relation to drawings of breasts (female), testes (male) and pubic hair (both male and female) development which were on a graphical scale from 1 (no development) to 5 (adult development).

### Statistical analysis

A single multilevel model was used to investigate the association between chronological and epigenetic ages. Using the multilevel model, we can include the measures of epigenetic age (as a repeated outcome) and calculate an intra-class correlation coefficient (ICC)—a number between 0 and 1 that suggests the proportion of variation (here in epigenetic age) which is explained by between-individual differences. The association between AA and developmental timing was assessed using Pearson correlation between AA (at birth, age 7 and age 17 years) and SITAR-estimated age at PHV. Multilevel models of the four ordinal Tanner stage variables, corrected for age at Tanner measurement, were used to assess the association of AA at birth and age 7 years on developmental timing. We also combined the pubic hair Tanner stage variables for boys and girls, and the breast/testes Tanner stage variables across boys and girls, in order to increase the power to detect an association with AA. Each model was adjusted for longitudinal cell composition estimated using the Houseman method.^27^

Body composition data were modelled using multilevel models,^28^^,^^29^ with AA (at birth and 7 years) included as a fixed effect along with an interaction of AA (at birth and age 7 years) with age to determine the effect of AA on changes in developmental characteristics. AA at age 17 years was not considered as an exposure, since it was recorded at the end the follow-up period, with few measures of the key traits occurring after it. In each multilevel model, we included sex, birth weight, gestational age, parity, delivery method, maternal age, maternal smoking, maternal alcohol consumption and maternal education level attained to adjust for potential confounding. Longitudinal cell counts (estimated using the Houseman method^27^) were also included, to adjust for the effect of changes in blood cell composition over the life course. To correct for temporality issues, only measures of development taken after AA were included in the multilevel models, e.g. AA at age 7 could only affect height measures after age 7 years. Weight was log-transformed to correct for non-constant variance over age (variance of weight increases over the life course). Cubic spline terms were used to account for the non-linear changes in height, log-weight and BMI. The placement of knots was based on previous research.^30–32^ For example, the multilevel model for height was:

$$\begin{array}{l} height_{ij}=\beta_{0i}+\beta_{\text{1}iageij}+\beta_{\text{2}}AA_{0}+\beta_{\text{3}}AA_{\text{7}}+\beta_{\text{4}}AA_{0}*age_{ij}\\ +\beta_{\text{5}}AA_{\text{7}}*age_{ij}+f_{i(ageij)}+\beta_{\text{6}sex}+\beta_{\text{7}parity}+\beta_{\text{8}birthweight}\\ +\beta_{\text{9}gestationalage}\;+\beta_{\text{1}0caesarean}+\beta_{\text{11}maternalage}+\beta_{\text{12}maternalsmoking}\\ +\beta_{\text{13}maternalalcohol}+\beta_{\text{14}maternaleducaiton}\;+\beta_{\text{15}CD8tCellsProp}\\ +\beta_{\text{16}CD4tCellsProp}+\beta_{\text{17}NaturalKillerCellsProp}+\beta_{\text{18}BcellsProp}\\ +\beta_{\text{19}MonocytesProp}+\beta_{\text{2}0GranulocytesProp}, \end{array}$$

where *height_ij_* is the *j*th height measurement from the *i*th individual for *i*=* *1, … , *n* individuals and *j*=1, … , *n_i_* measures. *β*_0__*i*_ and *β*_1__*i*_ represent the *i*th individual’s random intercept and slope; *f_i_* is a cubic spline which explains the height trajectory of individual *i*; *β*_2_ and *β*_3_ explain the association of age acceleration [at birth (*AA*_0_) and age 7 years (*AA*_7_), respectively] and average development; *β*_4_ and *β*_5_ explain the association of AA [at birth (*AA*_0_) and age 7 years (*AA*_7_), respectively] on changes in development; *β*_6_ to *β*_14_ describe associations between development and confounder variables; and *β*_15_ to *β*_20_ control for estimated cell composition.^27^

### Sensitivity analyses

We carry out two sensitivity analyses, modelling longitudinal physical development as above (A) with adjustment for age at puberty estimated using SITAR^26^ and (B) without adjusting for cell-type composition estimated using the Houseman method.^27^

## Results

**Figure 1.**
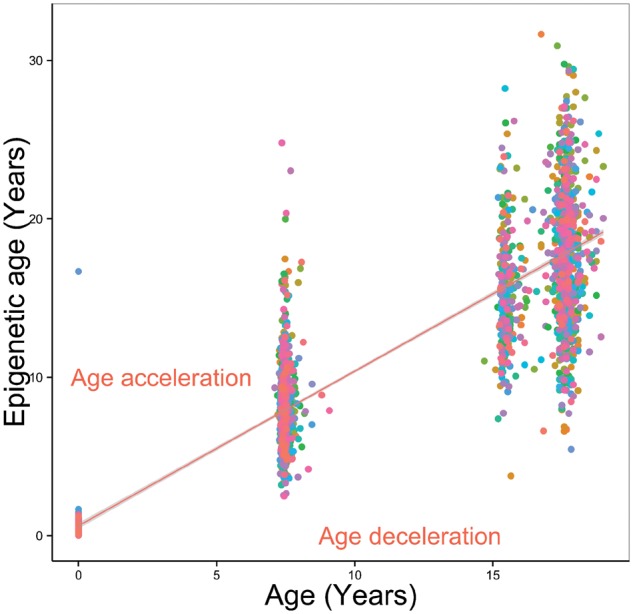
Epigenetic age against actual age for a random sample of 1000 ARIES offspring taken from across the three time points.

A summary of the cohort under investigation is given in Table 1. Epigenetic age at birth was 0.26 years on average; chronological age was lower than epigenetic age at the childhood time point (mean chronological 7.49, epigenetic 8.25) but similar at the adolescent time point (mean chronological 17.14, epigenetic 17.20). We find low Pearson correlation coefficients between chronological age and estimated age (*r* = 0.058 and 0.245 at childhood and adolescence, respectively); this reflects the low standard deviations in chronological age (SD = 0.15 in childhood, SD = 1.01 years in adolescence). High correlations (such as the *r* = 0.96 observed in the studies used to develop the measure of epigenetic age) were observed in data sets comprising a wide range of chronological ages.^2^ Correlations between estimated age and actual age are similar to the original Horvath paper when including data from across multiple time points; taking one random measure from each person, the correlation between epigenetic and actual age was 0.85 (Figure 1). Using a multilevel model including all measures of epigenetic and actual age, the coefficient of age was 0.985 [95% confidence interval (CI) 0.97, 1.00]. This suggests that, for each year of life, epigenetic age increases by 0.985 years on average. From this model, the intra-class correlation coefficient for epigenetic age was 0.12, which suggests that 12% of the variation in epigenetic age is between individuals.

**Table 1.** Characteristics of the ARIES sample

Variable	Time point	Mean	SD	Min.	Max.	*N* (%)
Age (years)	years	7.49	0.15	7.10	9.08	
	17 years	17.14	1.01	14.69	19.33	
DNA methylation age (years)	Birth	0.26	0.63	–0.59	16.68	
	7 years	8.25	2.42	2.50	24.80	
	17 years	17.20	4.34	3.77	31.65	
Height (cm)	7 years	126.24	5.29	109.20	141.60	
	17 years	171.93	9.11	152.20	197.50	
Weight (kg)	7 years	26.22	4.73	17.60	51.40	
	17 years	66.99	14.92	44.20	147.40	
BMI (kg/m^2^)	7 years	16.37	2.22	12.65	29.15	
	17 years	22.61	4.47	16.26	50.06	
BMD (g/cm^2^)	17 years	1.19	0.10	0.95	1.56	
Bone mass (g)	17 years	2814	547	1683	4666	
Fat mass (g)	17 years	18 005	11 478	3485	82 194	
Lean mass (g)	17 years	46 623	10 106	27 535	76 425	
Birth weight (g)		3418	547	645.00	5640	
Gestational age at delivery (weeks)		39.46	1.86	25.00	47.00	
Parity (# previous pregnancies)		0.7	0.8	0	5	
Maternal age at pregnancy (years)		29.2	4.4	17	42	
Sex	Male					445 (49)
	Female					469 (51)
Delivery method	Caesarean					83 (9)
	Natural					795 (91)
Maternal smoking in pregnancy	Never					545 (61)
	Quit					248 (28)
	Smoker					101 (11)

### AA at birth

AA was not associated with average length at birth (0.16 cm per year of AA, 95% CI –0.08, 0.39 cm; *P = *0.19) or height growth (0.017 cm/year per year of AA, 95% CI –0.067, 0.10 cm/year; *P = *0.69). There was evidence that children with higher AA at birth had faster growth in weight (0.25%/year faster growth per year of AA, 95% CI 0.034, 0.459%/year; *P = *0.023) and BMI (0.035 kg/m^2^/year faster growth per year of AA, 95% CI –0.0037, 0.066 kg/m^2^/year; *P = *0.030) during childhood and adolescence. There was little evidence for an association between AA at birth and either average BMD (0.003 g/cm^3^ per year of AA, 95% CI –0.006, 0.012 g/cm^3^; *P = *0.478) or bone mass (19.71g per year of AA, 95% CI –30.8, 70.2 g; *P = *0.45). A 1-year higher AA at birth was associated with 1321-g higher fat mass on average across childhood (95% CI 386, 2256 g; *P = *0.006), but this difference narrowed over time, with higher AA children having a slower growth of fat mass during childhood and adolescence (112.5 g/year slower growth, 95% CI 31, 194 g/year slower; *P = *0.007). AA at birth was not associated with average lean mass (–74.5g per year of AA, 95% CI –1502, 1353 g; *P = *0.918).

### AA in childhood

Higher AA at age 7 was associated with increased height (Table 2). Children with a 1-year higher AA at 7 were 0.23 cm taller on average (95% CI 0.04, 0.41 cm; *P = *0.018) between 7 and 17 years of age. AA at age 7 was also associated with changes in height, with a 1-year positive AA being associated with slower growth of height (–0.031 cm/year, 95% CI –0.005, –0.057 cm/year; *P = *0.021) from 7 to 17 years. There was no evidence of an association between AA at age 7 and either average weight (–0.11% per year of AA, 95% CI –0.69, 0.48%; *P = *0.72) or BMI (–0.04 kg/m^2^ per year of AA, 95% CI –0.11, 0.03 kg/m^2^; *P = *0.28). We did not identify any associations between AA at age 7 and either average BMD (–0.001 g/cm^3^ per year of AA, 95% CI –0.0036, 0.0015 g/cm^3^; *P = *0.418), bone mass (–7.16 g per year of AA, 95% CI –21.8 g, 7.5 g; *P = *0.34), fat mass (67.2 g per year of AA, 95% CI –205, 339 g; *P = *0.63) and lean mass (–206g per year of AA, 95% CI –605, 192 g; *P = *0.24).

**Table 2.** Age acceleration and physical development^a^

Outcome^b^	Exposure	Mean difference in outcome per 1-year greater AA	95% CI	*P*-value	Mean difference in change in outcome per year per 1-year greater AA	95% CI	*P*-value
Height (cm)	AA at 0	0.16	–0.08, 0.39	0.184	0.012	–0.071, 0.094	0.783
	AA at 7	0.23	0.04, 0.41	0.018	–0.031	–0.057, –0.005	0.021
Weight (%)^c^	AA at 0	–1.16	–2.86, 0.57	0.189	0.246	0.034, 0.459	0.023
	AA at 7	–0.11	–0.69, 0.48	0.719	–0.001	–0.072, 0.071	0.981
BMI (kg/m^2^)	AA at 0	–0.07	–0.18, 0.04	0.227	0.035	0.003, 0.066	0.030
	AA at 7	–0.04	–0.11, 0.03	0.282	0.004	–0.01, 0.01	0.423
BMD (g/cm^2^)	AA at 0	0.0032	–0.0056, 0.0119	0.478	–0.0002	–0.0010, 0.0006	0.600
	AA at 7	–0.0010	–0.0036, 0.0015	0.418	0.0001	–0.0001, 0.0003	0.298
Bone mass (g)	AA at 0	19.71	–30.83, 70.24	0.445	–0.66	–4.98, 3.66	0.765
	AA at 7	–7.16	–21.84, 7.51	0.339	1.07	–0.16, 2.31	0.089
Fat mass (g)	AA at 0	1320.8	385.85, 2255.7	0.006	–112.58	–194.39, –30.77	0.007
	AA at 7	67.26	–204.73, 339.24	0.628	–3.92	–27.30, 19.46	0.742
Lean mass (g)	AA at 0	–74.51	–1501.6, 1352.5	0.918	20.72	–80.98, 122.43	0.690
	AA at 7	–206.22	–605.36, 192.92	0.311	20.45	–7.77, 48.67	0.155

a All models adjusted for estimated cell counts, sex, birth weight, gestational age, parity, delivery method, maternal age, maternal smoking, maternal alcohol consumption and maternal education level attained.

b All outcome measurements come either concurrently or after the age at which AA is estimated.

c Weight was log-transformed such that back-transformed coefficients represent % change in weight.

### Role of age at puberty

AA at birth (Pearson *r = *0.006, *P = *0.85), 7 years (*r = *0.014, *P = *0.67) and 17 years (*r = *0.014, *P = *0.66) were not associated with age at PHV estimated by the SITAR model. The odds ratios from multilevel models of ordinal Tanner stages of development are presented in Table 3. Those boys with a positive epigenetic age at birth had higher odds of increasing Tanner stage of testes development (OR 1.10, 95% CI 1.01, 1.20; *P = *0.03). Further, combining across both sexes, there was some evidence that those children with positive epigenetic age at birth had higher odds of increasing pubic hair development in adolescence (OR 1.05, 95% CI 1.00, 1.11; *P = *0.06). There was no evidence that AA at age 7 was associated with any longitudinal Tanner measure of development.

**Table 3.** Results from multilevel ordinal models of Tanner stage variables against age acceleration at birth and age 7 years, controlling for age at measurement of Tanner stage

Outcome	Exposure	Odds ratio (per year of AA)	95% CI	*P*-value	*n*
Tanner girls genitals	AA at 0	1.11	0.87, 1.42	0.39	459
	AA at 7	0.99	0.73, 1.34	0.94	458
Tanner girls pubic hair	AA at 0	1.11	0.65, 1.88	0.70	410
	AA at 7	1.11	0.60, 2.07	0.74	415
Tanner boys genitals	AA at 0	1.10	1.01, 1.20	0.03	477
	AA at 7	1.04	0.94, 1.15	0.44	475
Tanner boys pubic hair	AA at 0	1.00	0.92, 1.07	0.90	448
	AA at 7	0.96	0.88, 1.05	0.37	453
Tanner genitals	AA at 0	1.00	0.92, 1.07	0.90	448
	AA at 7	0.96	0.88, 1.05	0.37	453
Tanner pubic hair	AA at 0	1.05	1.00, 1.11	0.06	925
	AA at 7	0.99	0.93, 1.06	0.79	928

### Sensitivity analysis

In Table 4, we provide the results of models that are adjusted for age at puberty, for comparison with Table 2. Whereas there is a general pattern of attenuation of the associations of AA with physical development after adjustment for age at puberty, there are no changes to the overall patterns of association described in the previous sections.

**Table 4.** Age acceleration and physical development with adjustment for age at puberty^a^

Outcome^b^	Exposure	Mean difference in outcome per 1-year greater AA	95% CI	*P*-value	Difference in average change in outcome per 1-year positive AA	95% CI	*P*-value
Height (cm)	AA at 0	0.17	–0.07, 0.40	0.167	0.009	–0.074, 0.092	0.828
	AA at 7	0.22	0.04, 0.41	0.019	–0.031	–0.058, –0.005	0.022
Weight (%)^c^	AA at 0	–0.95	–2.60, 0.72	0.262	0.198	0.001, 0.396	0.049
	AA at 7	–0.13	–0.69, 0.44	0.653	0.002	–0.064, 0.069	0.949
BMI (kg/m^2^)	AA at 0	–0.06	–0.18, 0.05	0.271	0.032	0.001, 0.063	0.042
	AA at 7	–0.04	–0.11, 0.03	0.245	0.005	–0.01, 0.01	0.356
BMD (g/cm^2^)	AA at 0	0.0026	–0.0063, 0.0115	0.565	–0.0002	–0.0010, 0.0006	0.617
	AA at 7	–0.0012	–0.0037, 0.0014	0.379	0.0001	–0.0001, 0.0003	0.268
Bone mass (g)	AA at 0	17.21	–34.08, 68.50	0.511	–0.68	–4.99, 3.63	0.756
	AA at 7	–7.93	–22.80, 6.93	0.295	1.03	–0.20, 2.27	0.101
Fat mass (g)	AA at 0	1253.7	325.44, 2182.0	0.008	–111.41	–191.95, –30.88	0.007
	AA at 7	40.43	–229.68, 310.54	0.769	–3.79	–26.88, 19.30	0.748
Lean mass (g)	AA at 0	–93.80	–1513.8, 1326.2	0.897	19.67	–81.48, 120.83	0.703
	AA at 7	–239.08	–636.16, 157.99	0.238	21.93	–6.13, 49.98	0.126

a All models adjusted for age at puberty, estimated cell counts, sex, birth weight, gestational age, parity, delivery method, maternal age, maternal smoking, maternal alcohol consumption and maternal education level attained.

b All outcome measurements come either concurrently or after the age at which AA is estimated.

c Weight was log-transformed such that back-transformed coefficients represent % change in weight.

Table 5 displays results unadjusted for longitudinal cell composition, as estimated by the Houseman method.^27^ Here, AA at age 7 appears to be associated with changes in both bone mass and lean mass. Associations between AA at 7 and height are similar with and without adjustment, as are all associations of AA at birth.

**Table 5.** Age acceleration and physical development without adjusting for cell type proportions^a^

Outcome^b^	Exposure	Mean difference in outcome per 1-year greater AA	95% CI	*P*-value	Mean difference in change in outcome per year per 1-year greater AA	95% CI	*P*-value
Height (cm)	AA at 0	0.17	–0.06, 0.40	0.142	0.011	–0.072, 0.093	0.802
	AA at 7	0.21	0.03, 0.40	0.025	–0.033	–0.059, –0.007	0.014
Weight (%)^c^	AA at 0	–0.99	–2.68, 0.74	0.260	0.233	0.018, 0.448	0.034
	AA at 7	–0.09	–0.67, 0.49	0.751	–0.002	–0.074, 0.071	0.966
BMI (kg/m^2^)	AA at 0	–0.08	–0.19, 0.03	0.151	0.035	0.004, 0.066	0.028
	AA at 7	–0.04	–0.10, 0.03	0.304	0.004	–0.01, 0.01	0.413
BMD (g/cm^2^)	AA at 0	0.0026	–0.0061, 0.0114	0.556	–0.0002	–0.0010, 0.0006	0.596
	AA at 7	–0.0015	–0.0040, 0.0010	0.235	0.0001	–0.0001, 0.0004	0.218
Bone mass (g)	AA at 0	14.79	–36.80, 66.38	0.574	–0.58	–4.97, 3.80	0.794
	AA at 7	–11.99	–26.50, 2.52	0.105	1.29	0.05, 2.54	0.042
Fat mass (g)	AA at 0	1289.8	355.91, 2223.7	0.007	–108.21	–190.09, –26.34	0.010
	AA at 7	81.73	–181.36, 344.83	0.543	–5.03	–28.25, 18.18	0.671
Lean mass (g)	AA at 0	–140.32	–1605.1, 1324.4	0.851	21.15	–82.88, 125.18	0.690
	AA at 7	–306.20	–708.80, 96.41	0.136	25.33	–3.32, 53.97	0.083

a All models adjusted for sex, birth weight, gestational age, parity, delivery method, maternal age, maternal smoking, maternal alcohol consumption and maternal education level attained.

b All outcome measurements come either concurrently or after the age at which AA is estimated.

c Weight was log-transformed such that back-transformed coefficients represent % change in weight.

## Discussion

Positive epigenetic AA in early life appears to be associated with several developmental variables and changes in these variables during childhood. We have identified positive associations between AA and average height, average fat mass, and increased weight and BMI gain. Conversely, there were negative associations between AA and changes in height and fat mass. A systematic difference between epigenetic and actual age at the ARIES childhood time point was found (mean actual 7.49 years, mean epigenetic 8.25 years). There may be population differences between the ARIES population and the cohorts of children used to develop the Horvath age estimation method. For example, the Alisch *et al.* data set^33^ has a higher proportion with non-European ancestry (>15%) and uses the Illumina 27k rather than 450k array to estimate epigenetic age. The systematic difference at childhood could further be influenced by the spread of the estimated epigenetic ages for the childhood time point (standard deviation 2.4 years, range 2.5–25 years) when compared with the spread of actual age at childhood (standard deviation 0.15 years, range 7.1–9.1 years).

The findings reported here are independent of sex (sex differences in AA have been previously reported^22^), with all analyses controlled for sex. Those children with higher AA at age 7 are taller on average with lower lean and bone mass. This suggests that there may be an identifiable developmental type, with higher AA in early life. Studies of AA in adults have identified a positive association between AA and obesity^10^ and all-cause mortality.^15^ Given that BMI and general adiposity are associated with an increased risk of mortality,^34^^,^^35^ this suggests an epigenetic age lower than one’s actual age (i.e. negative AA) is desirable. We have found some evidence to suggest that growth of BMI is faster in children whose DNA methylation levels at birth lead to a positive AA. This is congruent with several previous findings^10^^,^^22^ and suggests the link between AA and BMI manifests from birth. However, it is not yet clear whether positive AA is harmful during childhood. Indeed, it could be taken from our results that a positive AA suggests above-average development (which is not always a health positive, e.g. BMI). For example, we have also identified positive associations between AA and height and fat mass.

Whereas our study found at best a suggestive relationship between AA at birth and the role of sex hormones (Tanner stage), another study in adults found that the loss of sex hormones (resulting from menopause) was associated with increased epigenetic AA in blood.^14^ However, we did not identify any association between AA and age at puberty (estimated by age at PHV). One might expect that age at puberty (an obvious marker of developmental age) would be associated with epigenetic age but its inclusion in the modelling of development failed to influence the effect of AA. Further, a recent study of children who suffer from a severe developmental disorder found no evidence for a difference in epigenetic and chronological age.^23^ These null findings temper our conclusions on the relationship between AA and physical development. On the other hand, measurement error and tissue specificity may play a role. We used age at PHV (i.e. the age at which adolescents grow fastest) estimated by the SITAR model^26^ as a marker for age at puberty. Obtaining an accurate measure of age at puberty is difficult, and our null finding may be to do with poor estimates of age at puberty. Another possibility is that blood cells are not the optimal tissue for relating epigenetic age and physical development.

Future longitudinal studies of AA may be able to provide evidence as to the changing role of epigenetic age across the life course. Causal inference methods, such as Mendelian randomization,^36^ should be implemented to investigate the influence of epigenetic age and AA^37^ on development, perhaps using genetic variants close to the 353 CpG sites (these are described in our Supplementary data, available at *IJE* online) which are used to estimate epigenetic age. Since Mendelian randomization will require a large sample size to be adequately powered, collaboration between cohort studies with epigenetic and longitudinal data will be key to this endeavour.

A novel application of the epigenetic clock in physical development should involve the comparison of epigenetic age (and AA) between tissue types on the same individuals. Comparisons of epigenetic age of bone, blood and adipose tissue, for instance, could lead to novel insights into well-known associates of development and how they interact with changes across the life course. Another potential avenue is to use AA as an aggregate measure of development. Whereas our analysis has identified several associations, larger studies could identify stronger (and possibly causal) links between AA and development. Using AA as a marker for development would simplify analyses where difficulty lies in choosing which aspects of development to adjust for.

We have not been able to replicate our longitudinal analysis findings in an independent cohort due to the unique nature of our data set. Since measured cell-type proportions were not available in ARIES, we have adjusted for estimated cell-type proportions from the online calculator (http://labs.genetics.ucla.edu/horvath/dnamage/), which uses the Houseman method.^27^ This raises the possibility that differences observed can be explained by longitudinal (possibly developmental) changes in white blood cell profiles not captured by these estimates. Whereas adjusting for cell type is good practice, care should be taken when adjusting for cell composition in early life, since the Houseman method has not been validated in cord blood samples or in very young children and it may lead to biased results. In this manuscript, we have shown the results both adjusted and unadjusted for Houseman estimated cell counts. We observed that the association between epigenetic age and both bone and lean mass appears to be explained by changing cell-type composition across childhood and adolescence. However, this may be due to a bias introduced using the Houseman method on cord blood samples. Recently, reference data sets for cell-type correction in cord blood have been released.^38^^,^^39^ Unfortunately, using these in longitudinal modelling through childhood and adolescence is difficult, since these methods do not estimate the same cell types as those in venous blood drawn from the peripheral circulation.

Our main findings were obtained across seven multilevel models, each with two parameters of interest, and should thus be interpreted in light of this multiple testing burden. The association of AA with changes in height could be explained by regression to the mean. For instance, we find positive AA is associated with being taller on average at age 7, but also that positive AA is associated with slower growth from 7 to 17 such that, on average, children will end up with similar heights at age 17 regardless of AA.

Epigenetic AA in early life is associated with several developmental characteristics throughout childhood and adolescence, but with associations not all in the same direction, and no observed association with age at puberty. The consideration of epigenetic age as an index of developmental stage is a novel concept that adds to the growing literature around AA and its use as a measure of development aging. Further longitudinal and causal analyses are needed to investigate the influences and consequences of AA.

## Supplementary Data

Supplementary data are available at *IJE* online.

## Funding

This research was specifically funded by UK Economic & Social Research Council grant RES-060-23–0011; UK Medical Research Council grants G0601625, G0600705, MR/L011824/1 and MR/M020894/1; and European Research Council grant 269874. The UK Medical Research Council and the Wellcome Trust (Grant ref: 102215/2/13/2) and the University of Bristol provide core support for ALSPAC. ARIES was funded by the BBSRC (BBI025751/1 and BB/I025263/ 1). Supplementary funding to generate DNA methylation data which are (or will be) included in ARIES has been obtained from the MRC, ESRC, NIH and other sources. ARIES is maintained under the auspices of the MRC Integrative Epidemiology Unit at the University of Bristol (MC_UU_12013/2, MC_UU_12013/8 and MC_UU_12013/9). The MRC Integrative Epidemiology Unit receives funding from Sanofi for unrelated research.

**Conflict of interest:** The authors have no conflicts of interest to declare.

## Supplementary Material

Supplementary Data
